# The MS@Work study: a 3-year prospective observational study on factors involved with work participation in patients with relapsing-remitting Multiple Sclerosis

**DOI:** 10.1186/s12883-015-0375-4

**Published:** 2015-08-12

**Authors:** Karin van der Hiele, Dennis A. M. van Gorp, Marco A. P. Heerings, Irma van Lieshout, Peter J. Jongen, Michiel F. Reneman, Jac J. L. van der Klink, Frans Vosman, Huub A. M. Middelkoop, Leo H. Visser, on behalf of the MS@Work Study Group

**Affiliations:** 1 National Multiple Sclerosis Foundation, Mathenesserlaan 378, Rotterdam, 3023HB The Netherlands; 2 Department of Psychology, Section Health, Medical and Neuropsychology, Leiden University, PO Box 9555, Leiden, 2300 RB The Netherlands; 3 Department of Neurology, St. Elisabeth-TweeSteden Hospital, PO Box 90151, Tilburg, 5000 LC The Netherlands; 4 van Lieshout Arbo Advies, PO Box 325, Uden, 5400 AH The Netherlands; 5 MS4 Research Institute, Ubbergseweg 34, Nijmegen, 6522 KJ The Netherlands; 6 Department of Community & Occupational Medicine, University Medical Center Groningen, Ant. Deusinglaan 1, PO Box 30001, Groningen, 9713 AV The Netherlands; 7 University Medical Centre Groningen, Centre for Rehabilitation, University of Groningen, PO Box 30.002, Haren, 9750 RA The Netherlands; 8 Department of Social and Behavioral Sciences, Tranzo Scientific Center for Care and Welfare, Tilburg University, PO Box 90153, Tilburg, 5000 LE The Netherlands; 9 University of Humanistic Studies, PO Box 797, Utrecht, 3500 AT The Netherlands; 10 Department of Neurology, Leiden University Medical Centre, PO Box 9600, Leiden, 2300 RC The Netherlands

**Keywords:** Multiple Sclerosis, Work participation, Absenteeism, Cognitive abilities, Psychological factors, Fatigue, Physical disability

## Abstract

**Background:**

Multiple Sclerosis (MS) is the most common cause of neurological disability in young and middle-aged adults. At this stage in life most people are in the midst of their working career. The majority of MS patients are unable to retain employment within 10 years from disease onset. Leading up to unemployment, many may experience a reduction in hours or work responsibilities and increased time missed from work. The MS@Work study examines various factors that may influence work participation in relapsing-remitting MS patients, including disease-related factors, the working environment and personal factors.

**Methods/design:**

The MS@Work study is a multicenter, 3-year prospective observational study on work participation in patients with relapsing-remitting MS. We aim to include 350 patients through 15–18 MS outpatient clinics in the Netherlands. Eligible participants are 18 years and older, and either currently employed or within three years since their last employment. At baseline and after 1, 2 and 3 years, the participants are asked to complete online questionnaires (including questions on work participation, work problems and accommodations, cognitive and physical ability, anxiety, depression, psychosocial stress, quality of life, fatigue, empathy, personality traits and coping strategies) and undergo cognitive and neurological examinations. After six months, patients are requested to only complete online questionnaires. Patient perspectives on maintaining and improving work participation and reasons to stop working are gathered through semi-structured interviews in a sub-group of patients.

**Discussion:**

Prospective studies with long-term follow-up on work participation in MS are rare, or take into account a limited number of factors. The MS@Work study provides a 3-year follow-up on various factors that may influence work participation in patients with relapsing-remitting MS. We aim to identify factors that relate to job loss and to provide information about preventative measures for physicians, psychologists and other professionals working in the field of occupational health.

## Background

Multiple Sclerosis (MS) is a chronic, inflammatory, demyelinating and degenerative disease affecting the central nervous system [1]. MS is the most common cause of neurological disability in young and middle-aged adults [2, 3]. At this stage in life most people are at the beginning or in the midst of their working career. Work participation is important for a person’s sense of self-respect, social contacts and providing a feeling of usefulness and satisfaction. Job loss has been associated with worse self-reported health and increased adverse health behaviors after job loss [4]. A large number of patients with MS (55-58 %) are unable to retain employment following diagnosis [5, 6]. In those with a paid job, a reduction in hours or work responsibilities, presenteeism and increased time missed from work can be observed [7, 8]. From a societal perspective, the loss of productivity in MS leads to significant socio-economic costs. A recent Danish study highlighted the economic impact of costs associated with productivity loss in MS, which are much higher than the healthcare costs (including hospitalization costs, outpatient clinic costs, primary care costs and drug costs) [9]. In fact, employment rate was found to be affected up to eight years before the diagnosis of MS. In a Dutch study, it was found that production losses contribute to a significant proportion (45.8 %) of the total annual MS-related costs. It should be noted that these production costs are mainly related to early retirement due to MS, while costs related to long-term sick leave and short-term absence contribute less [10]. The high prevalence of unemployment among MS patients and the large personal and societal costs involved underline the need to study factors associated with unemployment and work absence in MS.

### Factors related to unemployment and work absence in MS

The causes of unemployment in MS involve a complex interaction between disease-related factors, the working environment, job demands and personal factors [11].

Many studies on work ability or work status in MS focus on physical and cognitive measures as primary determinants of work capacity. Cognitive functions such as working memory, episodic memory and processing speed have been identified as important predictors of employment status [12, 13]. Cognitive functioning remains an important predictor even when controlling for depression and disease course [12]. A 3-year follow-up of employed MS patients revealed that cognitive decline, especially in verbal memory and processing speed, distinguishes MS patients who progressed to work disability (28,9 %) from those who did not [14]. The latter study utilized both a conservative and liberal definition of deteriorated employment status. The conservative approach required a person to deteriorate from being gainfully employed to being disabled with formal disability benefits (occurring in 28,9 % of the participants over three years), while the liberal approach accepted any reduction in hours or work responsibilities (occurring in 45,5 % of the participants). A review study found ample support for the influence of physical functioning (indicated by level of neurological disability or the number and type of mobility aids) and cognitive functioning (indicated by memory functioning, verbal ability and self-perceived cognitive function) in work ability [15]. However, although limitations in physical and cognitive function hinder an individual’s ability to work, they cannot solely explain work capacity [15]. Other factors, such as educational level, type of work, fatigue, health perception, depression, personality and type of immunomodulatory treatment are also involved when predicting work participation [3, 8, 16, 17].

A study by Honarmand and colleagues [8] focused on the influence of neurological, demographic, psychological and cognitive factors on employment status (i.e. ‘employed’ versus ‘unemployed’) [8]. Cognitive and physical measures appeared as a robust predictor of employment status. Interestingly, higher depression scores and lower scores on the personality constructs ‘agreeableness’ and ‘extraversion’ were also associated with unemployment. A combination of symptoms of depression, less agreeableness and lower cognitive and physical functioning predicted 49,8 % of the variance in employment status.

The findings of a recent study indicate that already one year after the diagnosis of clinically isolated syndrome and relapsing-remitting MS, power of attention and memory are associated with a capability of working less hours, and that fatigue, depression and disease impact may negatively, and self-efficacy positively affect working hours [18].

The manner of coping with problems also tends to be a factor influencing work participation. Lode and colleagues [19] found that disability pensioned patients employed more problem-focused, emotion-focused and avoidant coping strategies than MS patients without a disability pension. Previous research has shown that emotion-focused and avoidant coping strategies are considered dysfunctional in adaptation to a chronic disease [20, 21].

The working environment is a very important factor for work participation when diagnosed with MS, including environmental factors, social factors, workplace factors and work demands [11]. Higher odds of unemployment have been observed in jobs requiring physical strength, manual precision and frequent moves, while protective factors include working in the public sector, a desk job or other sitting work [22]. Other important variables include accessibility and temperature of the workplace, available workplace accommodations (e.g. a flexible schedule, ergonomic work station, preferred parking), employer and colleague support, negative work events and perceived workplace difficulties [11, 23, 24].

Treatment with immunomodulators, including glatiramer acetate and the beta-interferons, is widely supported in relapsing-remitting MS [25]. Evidence suggests that immunomodulatory treatment reduces disease progression, as evidenced by a reduction in relapse rate [25] and less fatigue [26]. Several studies focused on the effects of immunomodulatory treatment on work absence in MS [16, 27, 28]. Type of immunomodulatory treatment was found to affect the number of days missed from work, although not always in the desired direction. Effects differed per type of immunomodulatory treatment.

### Study objectives and hypotheses

Most evidence for factors associated with work participation comes from cross-sectional studies. Prospective studies are rare, or take into account a limited number of factors. The main aim of the MS@Work study is to examine predictors of (changes in) employment status and work absenteeism in relapsing-remitting MS patients over a period of three years. By taking into account a wide variety of disease-related, work-related and personal factors this study may reveal typical patterns leading to changes in work participation. Identifying these patterns provides useful information about preventative factors for physicians, psychologists and other professionals working in the field of occupational health.

This leads to the following research questions:

1. Are there differences in cognitive, neurological and psychological functioning between employed and unemployed patients at baseline?
2. What (combination of) cognitive, neurological, psychological and work-related factors predict (changes in) employment status and work absenteeism over a period of three years?
3. What is the relation between initiating immunomodulatory treatment and work participation over a period of three years?
4. What reasons to stop working are provided retrospectively by patients with relapsing-remitting MS?
5. From a patient perspective: what can be done to maintain or improve work participation?

Overall, this study is considered exploratory; we include detailed cognitive, psychological and work-related measures that have not been studied in the context of longitudinal work participation. Employment status will be defined in both a conservative and liberal manner, and will also pertain to self-employed patients. The qualitative analyses will give more in-depth information about factors involved with work participation from a patient perspective.

Based on existing literature we expect to find more cognitive, physical and psychological problems in non-employed as compared with employed MS patients at baseline. Leading up to a lowered employment status and more work absenteeism, executive difficulties, increased fatigue, psychological problems and work difficulties are expected to become more apparent. MS patients receiving immunomodulatory treatment are expected to miss less days from work and have a more stable employment status over the 3-year study period, as compared with relapsing-remitting MS patients receiving no immunomodulatory treatment. This is expected to be related to less fatigue symptoms and relapses in patients treated with immunomodulators.

## Methods/design

### Design and setting

The MS@Work Study Group is a collaboration between health care professionals and researchers in the field of MS, occupational health professionals, and 15–18 MS outpatient clinics in the Netherlands. At this moment, a collaboration is set up with 15 MS outpatient clinics (Albert Schweitzer Ziekenhuis, Dordrecht, Alrijne Ziekenhuis Leiden, Leiden; Amphia Ziekenhuis, Breda; Canisius-Wilhelmina Ziekenhuis, Nijmegen; Catharina Ziekenhuis, Eindhoven; Elisabeth-TweeSteden Ziekenhuis, Tilburg; Jeroen Bosch Ziekenhuis,’s Hertogenbosch; Maasstad Ziekenhuis, Rotterdam; Medisch Centrum Alkmaar, Alkmaar; Medisch Centrum Leeuwarden, Leeuwarden; Orbis Medisch Centrum, Sittard; Rijnstate Ziekenhuis, Arnhem; St. Anna Ziekenhuis, Geldrop; St. Antonius Ziekenhuis, Nieuwegein and VieCuri Medisch Centrum, Venlo). The MS@Work study is a prospective, observational cohort study in 350 patients with relapsing-remitting MS who will be recruited from outpatient clinics in the Netherlands. Inclusion started in April 2014.

### Study population

Eligible subjects should be diagnosed with relapsing-remitting MS according to the Polman-McDonald criteria 2010 [29]. Furthermore, we plan to include patients that are 18 years and older, and either currently employed or within three years since their last employment. Patients with comorbid psychiatric or neurological disorders, substance abuse, neurological impairment that might interfere with cognitive testing (e.g. inadequate vision) or unable to speak and/or read Dutch are excluded from the study. Study visits will be rescheduled in case of an MS relapse within 1 month prior to the study visit.

### Sample size calculation

The main aim of the study is to examine predictors of (changes in) employment status and work absenteeism in relapsing-remitting MS patients over a period of three years. For this purpose a logistic regression analysis will be used. A power analysis based on a 5 % significance level, 80 % power, and an odds ratio of 2.1 indicates that at least 300 patients need to be included [30]. Taking into account a longitudinal drop-out rate of 10 % [31], the number of patients should be increased to a minimum of 330 patients. We aim to include at least 350 patients.

### Data collection, processing and storage

MS patients visiting outpatient clinics in the Netherlands, who meet the inclusion criteria, will be asked to participate in the MS@Work study. At baseline and after 1, 2 and 3 years participants will be asked to complete online questionnaires, and undergo neurological and cognitive examinations. After 6 months, participants only need to fill online questionnaires without undergoing neurological and cognitive examinations. Table 1 provides an overview of data collection per time point.

**Table 1:** Flowchart data collection

	Baseline	6 months	1 year	2 years	3 years
Informed consent	X	X (online)	X (online)	X (online)	X (online)
Online questionnaires	X	X	X	X	X
Neurological examination	X		X	X	X
Cognitive examination	X		X	X	X

The inclusion and exclusion criteria will be checked by a neurologist. Eligible patients will be asked to participate by a letter explaining the study procedures. The letter includes an informed consent form, a non-responder questionnaire and return envelope. Patients who are interested in participating are asked to send back the signed informed consent form. After receiving this form, the patient will be contacted and an appointment will be made for neurological and cognitive examinations. Patients are asked to complete several online questionnaires at home. For this purpose a personalized e-mail will be sent with a link to the online questionnaires. The questionnaires are to be completed within one week. Within this time frame the questionnaire may be filled in at moments that are suitable to the participant. Their answers will be saved automatically. In case the questionnaires have not been completed within one week, a reminder e-mail will be sent. Patients who do not have internet access, or who are not capable of using a PC are sent a paper-and-pencil version of the questionnaires. The neurological and cognitive examinations should be performed on one day, and should preferably take place at the outpatient clinic where the patient is treated.

The neurological examination will be performed by a neurologist or a neurologist in training. Psychologists or trained research nurses will perform the cognitive examinations. Patients will be offered a financial compensation for traveling expenses. With the patient’s approval, the general practitioner will be informed of the patient’s participation in the MS@Work study. The outpatient clinics will be asked to keep track of the number of patients that are invited to participate. Patients who do not wish to participate are asked to complete a brief non-responder questionnaire. This questionnaire will later be used to compare non-responders and responders on age, gender and job characteristics. A subgroup of 20 patients will be interviewed by one researcher to assess in a qualitative way the most important factors involved in employment from a patient perspective and (if applicable) their reasons for discontinuing employment.

The neurological and cognitive scoring forms are stored at the MS outpatient clinics. After completion of the study, the gathered data and scoring forms will be stored at the Faculty of Social Sciences (Leiden University) for a period of five years. An SPSS data file (IBM SPSS statistics 19) will be created in which the data are stored anonymously. Each patient will receive a personal code and any identifying information is removed from the source file. The source file will be accessible to the principal investigators upon request. Student assistants participating in data collection will be provided temporary access to the database. The key database and source file will both be safeguarded by the coordinating investigator.

### Materials/Measures

#### Neurological examination

In a structured interview data will be collected on disease course, relapse rate, time of last relapse, current and previously used medication, co morbid neurological and psychiatric disorders, substance abuse and neurological impairment that might interfere with cognitive testing (e.g. inadequate vision). The examination further includes the Expanded Disability Status Scale [32] and the Multiple Sclerosis Functional Composite [33] which combines the 9-Hole Peg Test [34] for assessing arm and hand function, the Timed 25-foot walk [35] for assessing mobility and leg function, and the Paced Auditory Serial Addition Test [36] (see cognitive examination).

#### Cognitive examination

An overview of the administered tests and corresponding cognitive domains is provided in Table 2. The cognitive examination consists of task representing cognitive domains like memory, processing speed, verbal word fluency, visual spatial processing and higher executive functioning. Most subtests of the Minimal Assessment of Cognitive Function in Multiple Sclerosis (MACFIMS) [12], the Trail Making Test [37] and subtests of the Delis-Kaplan Executive Function System [38] are used.

**Table 2:** Cognitive test battery

Test	Cognitive domain(s)
Visual Analogue Scale	Fatigue and energy (before and after examination)
Controlled Oral Word Association Test [41]	Verbal word fluency
Semantic Category Fluency [42]	Verbal word fluency
Judgment of Line Orientation Test [41]	Visual-spatial processing
Rey Verbal Learning Test [43, 44]	Learning and memory
Brief Visuospatial Memory Test-Revised [45]	Learning and memory
Symbol Digit Modalities Test [46]	Information processing speed and working memory
Paced Auditory Serial Addition Test [36]	Information processing speed, flexibility and working memory
Trail Making Test [37]	Information processing speed, attention and concept shifting and executive functioning
Delis-Kaplan Executive Function System Design Fluency Test [38]	Fluency in generating visual patterns, response inhibition and executive functioning
Delis-Kaplan Executive Function System Color Word Interference Test [38]	Information processing speed, attention and response inhibition and executive functioning

#### Questionnaires

The online questionnaires and the constructs they intend to measure are listed in Table 3. The Work Role Functioning Questionnaire and work-related questions in the general questionnaire are intended for participants with a paid job. The Multiple Sclerosis Neuropsychological Screening Questionnaire is intended for patients and informants. The Caregiver Strain Index and Neuropsychiatric Inventory are intended for the caregiver or informant. The NEO Five-Factor Personality Inventory is only administered at baseline. In working-age adults the big-five personality traits are considered stable over a four-year period [39].

**Table 3:** Questionnaires

Questionnaire	Measures
General questionnaire	Demographics, disease characteristics, medication, characteristics of current and previous jobs, absenteeism, presenteeism, work ability [47], work-related problems and accommodations [23] and important job aspects.
Work Role Functioning Questionnaire [48]	Health-related work functioning
Multiple Sclerosis Neuropsychological Screening Questionnaire [49]	Cognitive impairment
Hospital Anxiety and Depression Scale [50]	Depression and anxiety
Symptom Checklist-90-R (sleep difficulties) [51]	Sleep difficulties
Modified Fatigue Impact Scale-20 [52]	Fatigue impact
Checklist Individual Strength-20-R [53]	Fatigue
Every Day Problem Checklist [54]	Chronic everyday stress
Multiple Sclerosis Quality of Life-54 [55]	Quality of life
NEO Five-Factor Personality Inventory [56]	Personality
Coping Inventory for Stressful Situations [57]	Coping strategies
Empathy Quotient [58]	Empathy
Multiple Sclerosis Work Difficulties Questionnaire [24]	Work difficulties
Caregiver Strain Index [59]	Caregiver Strain
Neuropsychiatric Inventory [60]	Neuropsychiatric problems

### Statistical analyses

Statistical analyses will be performed using SPSS 21.0 (Statistical Package for the Social Sciences version 21.0, IBM, Chicago, Illinois, USA). ATLAS.ti (ATLAS.ti Scientific Software Development GmbH, Berlin, Germany) will be used to perform qualitative data analyses.

#### Predictors of work participation

Depending on the distribution of the data, independent t-tests, analysis of covariance (ANCOVA), Mann–Whitney U and Chi-square tests will be used to examine baseline differences in cognitive, neurological and psychological variables (dependent variables) between employed and unemployed relapsing-remitting MS patients (independent variable). Logistic regression analyses will be used to study predictors (cognitive, neurological, psychological and work-related factors) of employment status (i.e. employed versus unemployed/deteriorated versus stable) in relapsing-remitting MS patients with a paid job after 1, 2 and 3 years. Multiple regression analysis will be performed to study associations between cognitive, neurological, psychological and work-related factors (independent variables) and work absenteeism (dependent variable) in relapsing-remitting MS patients with a paid job after 1, 2 and 3 years. Repeated measures analysis of variance will be used to examine cognitive, neurological and psychological changes over a period of three years (within-subject factor) in relapsing-remitting MS patients who lose employment, versus those who retain employment, versus those who remain unemployed (between-subjects factor).

#### Immunomodulatory treatment and work participation

Treatment naive patients initiating treatment with an immunomodulator (i.e. interferon beta-1a, interferon beta-1b and glatiramer acetate) will be compared with treatment naive relapsing-remitting MS patients not initiating immunomodulatory treatment. Treatment naive in this context is defined as not previously treated with an immunomodulatory treatment. Depending on the distribution of the data, independent t-tests, ANCOVA, Mann–Whitney U, and Chi-square tests will be used to examine differences in employment status and work absenteeism (dependent variables) between patients initiating immunomodulatory treatment and patients not initiating this type of treatment (independent variable). Tests will be performed after 6 months, 1, 2 and 3 years. A paired samples *t*-test will be used to compare work absenteeism before and after starting immunomodulatory treatment after 6 months, 1, 2 and 3 years. Separate analyses will be performed for each type of immunomodulatory treatment. On an exploratory basis, we will examine changes in fatigue and physical disability before and after treatment.

#### Qualitative analyses of semi-structured interviews

To access meanings, structures and essences of employment 20 semi-structured interviews will be conducted. The research design moves from lower-level analysis to higher-level theorizing. As research-strategy, Grounded Theory will be used. For detailed exploration of the data, quantitative analysis and Interpretative Phenomenological Analysis (IPA) will be used.

### Ethical considerations

The study was approved by the Medical Ethical Committee Brabant (NL43098.008.12 1307) and the Board of Directors of the participating MS outpatient clinics. The study will be conducted according to the principles of the Declaration of Helsinki (amended by the 64th WMA general Assembly, Fortaleza, Brazil, October 2013) and in accordance with the Medical Research Involving Human Subjects Act (WMO) [40].

## Discussion

The MS@Work study provides a 3-year follow-up on various factors that may influence work participation in patients with MS. Key strengths of the study are its multi-factorial approach and longitudinal design. We include several disease-related, work-related and personal factors, that have rarely been studied in combination or longitudinally. By examining the patients’ physical and mental well-being and their (work) situation at regular time intervals, we aim to reveal typical patterns leading to changes or stability in work participation. This can be viewed in terms of job loss, but also in terms of which conditions should be met to maintain a job or to attain one. Another strength is our use of different (operational) definitions of work participation and related constructs, such as work ability, work-related problems, important job aspects and the need for accommodations.

Qualitative data analyses will be used to examine patient perspectives on what can be done to improve work participation and reasons to stop working. This may provide unique insights in the current socio-economic climate, that may or may not be beneficial for work participation in patients with a chronic disease such as MS. Potential study limitations include a selection bias; only relapsing-remitting MS patients are included who visit outpatient clinics in the Netherlands. We specifically chose to include patients with a relapsing-remitting disease course, as these are generally ambulatory and there is a higher possibility of work participation. This study will be carried out in a Dutch health care and social security context. Generalization of results to other contexts may be limited.

More than half of the people diagnosed with MS are unable to retain employment which is associated with high socioeconomic costs. Combined with the fact that work participation is not only important for a person’s financial security, but also for one’s sense of self-respect, social engagement and feelings of usefulness, the need to research factors influencing work participation in MS is eminent. The MS@Work study provides an excellent starting point for future research on work participation in MS. As the study aims to provide information about preventative factors for job loss, the outcomes may be used to design job training programs for MS patients and to design effective strategies to inform both patients and employers how to help MS patients maintain their jobs.
